# Surface‐Enhanced Raman Scattering Imaging Assisted by Machine Learning Analysis: Unveiling Pesticide Molecule Permeation in Crop Tissues

**DOI:** 10.1002/advs.202405416

**Published:** 2024-06-24

**Authors:** Xiaotong Wang, Xiaomeng Sun, Zhehan Liu, Yue Zhao, Guangrun Wu, Yunpeng Wang, Qian Li, Chunjuan Yang, Tao Ban, Yu Liu, Jian‐an Huang, Yang Li

**Affiliations:** ^1^ State Key Laboratory of Frigid Zone Cardiovascular Diseases (SKLFZCD), Research Center for Innovative Technology of Pharmaceutical Analysis College of Pharmacy Harbin Medical University Heilongjiang 150081 P. R. China; ^2^ Research Unit of Health Sciences and Technology (HST) Faculty of Medicine University of Oulu Oulu 999018 Finland; ^3^ Department of General Surgery, The Fourth Affiliated Hospital of Harbin Medical University, and Department of Pharmacology (State Key Laboratory of Frigid Zone Cardiovascular Diseases, Ministry of Science and Technology; The Key Laboratory of Cardiovascular Research, Ministry of Education) at College of Pharmacy Harbin Medical University Baojian Road, Nangang District Harbin 150081 P. R. China; ^4^ Department of Clinical Laboratory Diagnosis, Fourth Affiliated Hospital of Harbin Medical University Harbin Medical University Baojian Road, Nangang District Harbin 150081 P. R. China; ^5^ College of Bioinformatics Science and Technology Harbin Medical University Heilongjiang 150081 China

**Keywords:** 3D and dynamic SERS imaging, artificial intelligence, perovskite/silver nanoparticles composites, pesticide penetration, surface‐enhanced Raman scattering imaging

## Abstract

Surface‐enhanced Raman scattering (SERS) imaging technology faces significant technical bottlenecks in ensuring balanced spatial resolution, preventing image bias induced by substrate heterogeneity, accurate quantitative analysis, and substrate preparation that enhances Raman signal strength on a global scale. To systematically solve these problems, artificial intelligence techniques are applied to analyze the signals of pesticides based on 3D and dynamic SERS imaging. Utilizing perovskite/silver nanoparticles composites (CaTiO_3_/Ag@BONPs) as enhanced substrates, enabling it not only to cleanse pesticide residues from the surface to pulp of fruits and vegetables, but also to investigate the penetration dynamics of an array of pesticides (chlorpyrifos, thiabendazole, thiram, and acetamiprid). The findings challenge existing paradigms, unveiling a previously unnoticed weakening process during pesticide invasion and revealing the surprising permeability of non‐systemic pesticides. Of particular note is easy to overlook that the combined application of pesticides can inadvertently intensify their invasive capacity due to pesticide interactions. The innovative study delves into the realm of pesticide penetration, propelling a paradigm shift in the understanding of food safety. Meanwhile, this strategy provides strong support for the cutting‐edge application of SERS imaging technology and also brings valuable reference and enlightenment for researchers in related fields.

## Graphical abstract

Artificial intelligence techniques are presented as the first‐ever application to analyze images, pioneering an innovative, visualization strategy for pesticide penetration based on 3D and dynamic SERS imaging with perovskite/silver nanoparticles composites (CaTiO_3_/Ag@BONPs). The permeation of a series of pesticides (chlorpyrifos, thiabendazole, thiram, and acetamiprid) is investigated and quantitatively analyzed to achieve rapid identification and prediction of pesticide spectra.

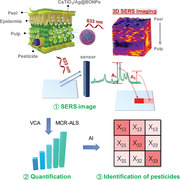

## 1 Introduction

In modern agriculture, pesticides applied to fruits and vegetables may be translocated through plant vascular systems from the site of application to other parts,^[^ ^1^, ^2^, ^3^, ^4^ ^]^ which directly endangers human health during consumption. Therefore, it is urgent to explore the penetration behavior of pesticides in fruits and vegetables in order to promote healthy food consumption and better guide the scientific application of pesticides. Nevertheless, due to the complex pulp composition, coupled with the influence of fluorescence interference as well as other impurities, making the information of pesticides is not easy to obtain. More importantly, pesticide molecules can enter fruit flesh and persist to be difficult to remove.^[^ ^5^ ^]^

Surface‐enhanced Raman scattering (SERS) based on plasmonic nanostructures has served as a powerful analytical tool for monitoring trace drug molecules owing to fingerprinting patterns for multi‐molecule analysis.^[^ ^6^, ^7^ ^]^ There exists SERS combined with microneedle patch method for detecting pesticide residues both inside and outside fruits and vegetables,^[^ ^8^ ^]^ however, the visualization of pesticide penetration has not been realized. With the increasing attention of SERS imaging technology in the field of scientific research, it has shown excellent performance advantages compared with traditional Raman imaging in providing ultra‐high molecular detection sensitivity, extremely low background noise, and in‐depth molecular level information.^[^ ^9^, ^10^ ^]^ Although permeable nanoparticles can be utilized for imaging and observing pesticide residues within fruits and vegetables, the observation range is limited by the penetration depth of nanoparticles, thus internal pesticides cannot be effectively eliminated.^[^ ^11^, ^12^ ^]^ SERS imaging technique also faces significant technical bottlenecks in ensuring balanced spatial resolution, preventing image bias induced by substrate heterogeneity, accurate quantitative analysis, and substrate preparation that enhances Raman signal strength on a global scale.

To systematically solve these problems, an innovative enhanced substrate for SERS imaging is designed, which takes the lead in integrating machine learning technology to develop an accurate quantitative analysis strategy for SERS images. When perovskite is introduced into silver nanosystem, the molecules adsorbed on its surface can form new charge‐transfer states at the interface. Meanwhile, perovskite as a dielectric media can retard the attenuation of transient surface plasma and electromagnetic waves to generate an intensive electromagnetic field for the enhancement of SERS signal, and obtain superposition effect greatly increasing SERS signal of pesticides.^[^ ^13^, ^14^, ^15^, ^16^, ^17^ ^]^ Artificial intelligence (AI) technology can instantaneously analyze the large amount of data covered in SERS imaging to obtain pesticide penetration information. Vertex Component Analysis (VCA), multivariate curve resolution alternate least squares (MCR‐ALS), and Euclidean distance (ED) are powerful tools for SERS imaging data analysis, which can eliminate the interference of other complex components in pulp and provide quantitative information for accurately analyzing the distribution of pesticides during SERS imaging.^[^ ^18^, ^19^, ^20^, ^21^, ^22^, ^23^, ^24^ ^]^ In addition, K‐means cluster analysis method in spatial machine learning (SML) technique has the ability to reveal the spatial information of pesticide penetration, predicting the types of pesticides present in fruits and vegetables by training the ability of the model to identify pesticides.^[^ ^25^ ^]^

**Figure 1:**
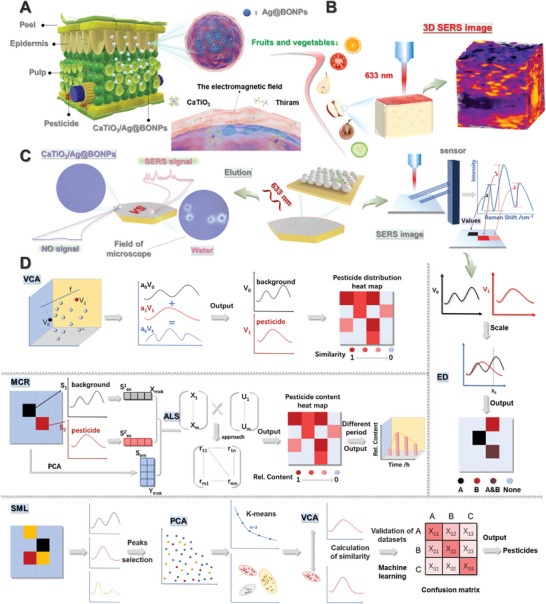
Schematic diagram illustrating the research on pesticide penetration behavior. A) The distribution of penetrated pesticides and sprayed CaTiO_3_/Ag@BONPs on plant profiles. B) The schematic diagram of 3D SERS imaging. C) Verification of the ability of CaTiO_3_/Ag@BONPs to detect, adsorb and remove pesticides, and interpretation of the SERS imaging process for pesticides in fruits and vegetables. D) The principle and process of artificial intelligence analysis methods, including vertex component analysis (VCA), multivariate curve resolution‐alternating least squares (MCR‐ALS), Euclidean distance (ED), and spatial machine learning (SML).

Here, artificial intelligence analysis combined with 3D and dynamic SERS imaging based on multifunctional perovskite/silver nanoparticles composites was utilized to propose the visual in situ pesticide penetration monitoring strategy (CaTiO_3_/Ag@BONPs‐SERS‐AI, **Figure** **1**). The penetration and amount of organophosphorus (chlorpyrifos), benzimidazole (thiabendazole), thiocarbamate (thiram), and chlorinated nicotinoid (acetamiprid) pesticides in pulp were investigated, and the detection and cleaning capability of CaTiO_3_/Ag@BONPs for pesticide residues was verified (Figure 1A,C). The spatial distribution of pesticides in fruits was clearly demonstrated by 3D SERS imaging (Figure 1B). Meanwhile, the penetrability difference between mixed and single applications of different pesticides was also studied. Apart from that, multivariate statistical learning methods were applied to process the imaging results, and a spectral recognition and classification model was established through machine learning to screen a large number of spectral data (Figure 1D). This strategy is the first application of novel nanomaterials in an attempt to obtain a pesticide penetration pattern that differs from previous perceptions. Scientific research on the principle of pesticide penetration can provide practical guidance for agricultural applications. The innovative strategy delves into the realm of pesticide penetration and also provides strong support for the cutting‐edge application of SERS imaging technology.

## 2 Results and Discussion

### 2.1 Characterization and Multifunctional Verification of CaTiO_3_/Ag@BONPs

**Figure 2:**
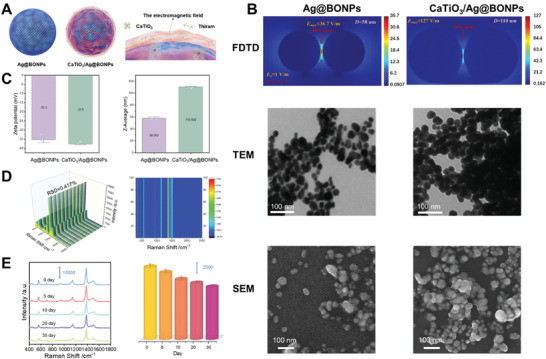
Characterization of substrate. A) The preparation process of CaTiO_3_/Ag@BONPs. B) The electromagnetic field simulation of the Finite Difference Time Domain (FDTD) of thiram, the transmission electron microscope (TEM), and scanning electron microscope (SEM) by Ag@BONPs and CaTiO_3_/Ag@BONPs. C) The dynamic light scattering (DLS) of Ag@BONPs and CaTiO_3_/Ag@BONPs. D) SERS spectra obtained by 100 randomly selected groups of thiram using the current method. E) Stability verification of thiram spectra and intensity change histogram of 1380cm^‐1^ characteristic peak within 30 days.

Silver nitrate was reduced by an appropriate amount of sodium borohydride (Ag@BONPs) to dynamically maintain the enhanced activity and stability of the substrate and prolong its service time.^[^ ^26^, ^27^ ^]^ As depicted in Figure S1 (Supporting Information), no other interferences and Raman signals were observed in the CaTiO_3_/Ag@BONPs system and thiram solution, respectively. However, an obvious characteristic fingerprint of thiram was obtained by CaTiO_3_/Ag@BONPs, showing an extremely high signal‐to‐noise ratio (*EF* = 2.8 × 10^8^, Text S1, Supporting Information). Each band in the spectrum of pesticides was assigned and listed in Table S1 (Supporting Information). Arbitrary SERS spectra of 100 sets of thiram at different time periods were measured, and the RSD value of the characteristic Raman shift intensity at 1380 cm^−1^ was 0.417%, showing superior reproducibility (**Figure** **2**D). In the SERS spectra, some characteristic peaks experienced a lesser extent shift, which might be due to the interaction between pesticides and the components in silver nanoparticles and apples.^[^ ^28^ ^]^

The silver nanoparticles before and after addition of CaTiO_3_ were characterized and evaluated by transmission electron microscopy (TEM), scanning electron microscopy (SEM), dynamic light scattering (DLS), and X‐ray diffraction (XRD), as shown in Figure 2B,C and Figure S2 (Supporting Information). The finite difference time domain (FDTD, Figure 2B) method was further applied to simulate the electromagnetic field distribution and explore the theoretical mechanism of signal enhancement (Text S2, Supporting Information). The simulation results show that the *EF*_EM_ value of Ag@BONPs reaches 1.8 × 10^6^, while the *EF*_EM_ value of CaTiO_3_/Ag@BONPs reaches 2.6 × 10^8^, which is consistent with the experimental results. SEM and SERS imaging characterization of CaTiO_3_/Ag@BONPs distribution in the spraying state were shown in Figures S3 and S5 (Supporting Information), and the corresponding FDTD simulation was performed according to the substrate distribution (Figure S4, Supporting Information). The elemental distribution of CaTiO_3_/Ag@BONPs was analyzed using energy dispersive spectrometer (EDS) mapping (Figure S6, Supporting Information), which can be observed that the perovskites were uniformly distributed in the system. The stability of CaTiO_3_/Ag@BONPs substrate was evaluated within 30 days and showed good stability (Figure 2E). The effect of CaTiO_3_/Ag@BONPs on cucumber longevity was monitored by tracking chlorophyll content, which reflects plant senescence or apoptosis. The chlorophyll content of cucumber sprayed with CaTiO_3_/Ag@BONPs was similar to that of the blank control (Figure S7, Supporting Information).

**Figure 3:**
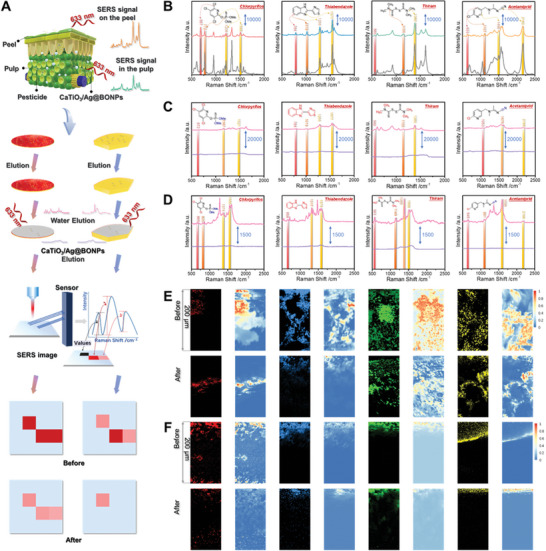
Demonstration of the ability of CaTiO_3_/Ag@BONPs to detect and adsorb pesticides. A) Schematic representation of pericarp and pulp detection. B) SERS signals of chlorpyrifos (red line), thiabendazole (blue line), thiram (green line), and acetamiprid (yellow line) in pulp compared with SERS signals (grey line) on the pericarp. SERS signals of chlorpyrifos, thiabendazole, thiram, and acetamiprid were obtained on pericarp C) and in the pulp D) after washing with water (pink line) compared with CaTiO_3_/Ag@BONPs (purple line). Red and blue colors indicate the lipophilic group and hydrophilic group, respectively. SERS imaging (black background) and corresponding VCA analysis (blue background) before and after CaTiO_3_/Ag@BONPs washed chlorpyrifos (red), thiabendazole (blue), thiram (green), and acetamiprid (yellow) on pericarp E) and in the pulp F).

A total of four kinds of pesticides were evaluated, as depicted in **Figure** **3**A,B, and the signature signals of pesticides on pericarp and in the pulp 24 h after pesticide spraying could be clearly observed, which was consistent with the Raman signal of pesticide solid standard (Figure S8, Supporting Information). CaTiO_3_/Ag@BONPs was further applied to detect trace pesticides, and the detection limits were all lower than pesticide maximum residue level values in food (Figure S9, Supporting Information). This result proved that CaTiO_3_/Ag@BONPs could obtain excellent signals in pesticide detection in peel and complex pulp tissue, and was superior to the traditional detection method (Figures S10 and S11, Supporting Information), which could be widely applied in pesticide residue detection.

In addition to assessing the sensitivity of the substrate to the samples in complex environments, other properties were evaluated. In the two parallel experimental groups, the apples were sprayed with pesticides under the same environment, and after natural drying, the pesticide signal of peel (Figure 3C) and pulp (Figure 3D) after washing with water and CaTiO_3_/Ag@BONPs were observed separately, and the detailed procedure was shown in Figure S12 (Supporting Information). The signals of pesticides became weak, and the pesticide signal after CaTiO_3_/Ag@BONPs washing was weaker than that after water washing. The elution efficiency of CaTiO_3_/Ag@BONPs was further measured by performing SERS imaging and VCA analysis, as shown in Figure 3E,F, which clearly revealed that CaTiO_3_/Ag@BONPs could clean and remove the pesticide from the pericarp and pulp. This result could also be verified by microscopy (Figure 1C), after the pesticide was cleaned with the substrate, the microscope field was dark and no pesticide signal was detected. However, after cleaning with water, the field of view is brighter and pesticide signal was displayed, indicating that CaTiO_3_/Ag@BONPs was even expected to be an “indicator” of pesticide distribution to visually display the distribution of pesticides.

The above demonstrated that CaTiO_3_/Ag@BONPs can not only detect a variety of pesticides in the pulp but also have cleaning properties for pesticides because it is based on the principle of similar phase solubility, water has a stronger cleaning ability for water‐soluble pesticides but a slightly weaker cleaning ability for fat‐soluble pesticides, which were consistent with the polarity of pesticides. Furthermore, CaTiO_3_/Ag@BONPs directly combine with pesticides to form new chemical bonds,^[^ ^29^ ^]^ which are not limited by the characteristics of pesticides and have a superior adsorption capacity for pesticides with various structures. CaTiO_3_/Ag@BONPs could maintain high sensitivity of detection samples in a complex environment, have a certain degree of fluorescence quenching effect, and have superior pesticide adsorption ability.

### 2.2 Dynamic Detection Strategy of Pesticide Penetration Based on SERS Imaging Combined with VCA and MCR‐ALS Data Processing Methods

**Figure 4:**
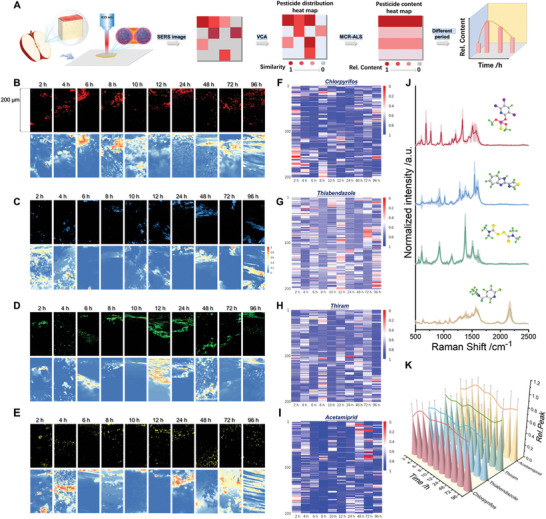
Pesticide penetration behavior and content analysis of pesticides in apples. A) Schematic representation of SERS imaging and data processing principles. B–E) Pesticide penetration SERS imaging (black background) detection and corresponding VCA analysis (blue background) of chlorpyrifos (red), thiabendazole (blue), thiram (green), and acetamiprid (yellow) at 2, 4, 6, 8, 10, 12, 24, 48, 72, and 96 h. F–I) The heat maps of penetration content of chlorpyrifos, thiabendazole, thiram, and acetamiprid were analyzed by MCR‐ALS at 2, 4, 6, 8, 10, 12, 24, 48, 72, and 96 h. J) Representative SERS spectra. The center lines represent a mean of ten spectra from one apple pulp sample to show general signal patterns. The light‐colored area represents the standard deviation range. K) The change of penetration content of chlorpyrifos, thiabendazole, thiram, and acetamiprid was obtained at 2, 4, 6, 8, 10, 12, 24, 48, 72, and 96 h. Colored lines represent mean changes obtained from a B‐spline curve fit.

As an eminent detection substrate, CaTiO_3_/Ag@BONPs could be used for a comprehensive study of pesticide penetration behavior, likewise, SERS imaging technology played a great role in simulating the actual pesticide spraying environment. **Figure** **4**A shows the pesticide penetration imaging process in apples and related data analysis principles. After pesticide spraying (chlorpyrifos, thiabendazole, thiram, and acetamiprid) on the surface of apples, the pesticide characteristic peaks in apple pulp within 200 µm at 2, 4, 6, 8, 10, 12, 24, 48, 72, and 96 h, respectively, were detected using SERS imaging (Figure 4J). The imaging results are depicted in Figure 4B–E (black background), which also shows the penetration distribution of different pesticides. However, since the fluorescence was too high, it might confuse the target components and interfering components, resulting in inaccurate results.^[^ ^30^ ^]^ Therefore, the imaging results were analyzed by VCA to distinguish the background. Later, the SERS map was transferred to the heat map to obtain the distribution of pesticides in the pulp accurately. The color of the heat map represents the association between the spectrum extracted from the pulp and the characteristic fingerprint spectrum of pesticides. The more similar the spectrum was to pesticide, the closer the color was to red, and conversely, the closer it was to blue. It could be seen from the corresponding heat map in Figure 4B–E (blue background) that the distribution was similar to that of imaging, and the two methods were mutually verified to reliably represent the distribution of pesticide in the pulp. The specific distribution of different pesticides in the pulp at different times was observed, and the penetration of these four pesticides at different times varied greatly. It could be seen from the distribution of pesticides in imaging that at the beginning of penetration, the penetration depth gradually increased with time. However, with the continuous increase in time, the pesticide signal penetration decreased, and the pesticide signal was observed again near the epidermis. This might be caused by the protective reaction of apples against pesticide penetration,^[^ ^31^ ^]^ where pesticides were gradually broken down by enzymes, their structures were changed or were broken down into inorganic substances, such as H_2_O with no Raman activity or CO_2_,^[^ ^32^, ^33^, ^34^ ^]^ showing low penetration depth. While with the gradual consumption of enzymes, the decomposition of pesticides slowed down, and the pesticides continued to penetrate deeper until enough pesticides were decomposed by enzymes.

MCR‐ALS was applied to further decompose and describe the multidimensional spectral data in the above VCA analysis results to explore the penetration degree of pesticides in the pulp. The contents of pesticides in different penetration degrees were analyzed, then the penetration of pesticides at different times was compared horizontally to obtain more accurate pesticide penetration information. The intensity of pesticide characteristic peaks at the same micron was averaged to plot the heat map. As depicted in Figure 4F–I, the pigment blocks in the heat map represent the pesticide content at the corresponding depth. The higher the content, the closer the color was to blue, whereas the lower the content, the closer it was to red. The results indicated that the relative content was higher for acetamiprid, while the relative content was less for thiabendazole.

Furthermore, the MCR‐ALS results in SERS imaging of the same time period were averaged to draw the content change curve, and the total change of pesticide penetration in different time periods was analyzed to quantify pesticide penetration on plant profiles, as depicted in Figure 4K and Figure S13 (Supporting Information). It can be seen that the trend of relative total penetration content of different pesticides was consistent with the results in Figure 4F–I. The difference in permeation of the above‐mentioned pesticides at the same concentration was due to different chemical structures of the pesticides. The transmission of pesticides in plants has two ways: lipophilic and hydrophilic.^[^ ^35^, ^36^, ^37^ ^]^ Lipophilic pesticides are more permeable than hydrophilic pesticides, but lipophilic pesticides could be blocked to a certain extent by the endodermal cortex while penetrating the epidermis.^[^ ^38^, ^39^ ^]^ The above pesticides are mostly lipophilic pesticides; of which, thiram contains four lipophilic methyl groups, and thiabendazole contains strong lipophilic thiazole groups. In addition to a lipophilic halogen group, acetamiprid also has a hydrophilic cyano group, which allows it to continue to permeate into the plant through the hydrophilic pathway even when the lipophilic pathway is weakened during penetration process. Coupled with the fact that acetamiprid is a systemic pesticide, may allow acetamiprid to permeate into the pulp in maximum quantities. Similarly, chlorpyrifos has three lipophilic halogen groups and two weak hydrophilic methoxy groups, which are able to penetrate into crops in small amounts via a hydrophilic pathway. This may be due to the fact that thiabendazole and thiram have only lipophilic functional groups, their permeability is low, and the difference is not significant. These results confirmed that both systemic and non‐systemic pesticides could penetrate into crops to varying degrees. In terms of apple cultivation, benzimidazole fungicides such as thiabendazole can be considered, and other insecticides can be collocated according to the situation as necessary. Acetamiprid and chlorpyrifos, with relatively fast and large penetration rates in systemic pesticides and non‐systemic pesticides, were selected as the representatives for subsequent penetration monitoring in various fruits and vegetables. In order to exhibit the process of pesticide penetration more intuitively, dynamic SERS imaging of acetamiprid and chlorpyrifos was collected (Movies S1 and S2, Supporting Information).

### 2.3 Application of Dynamic Detection Strategy of Pesticide Penetration in Fruits and Vegetables

**Figure 5:**
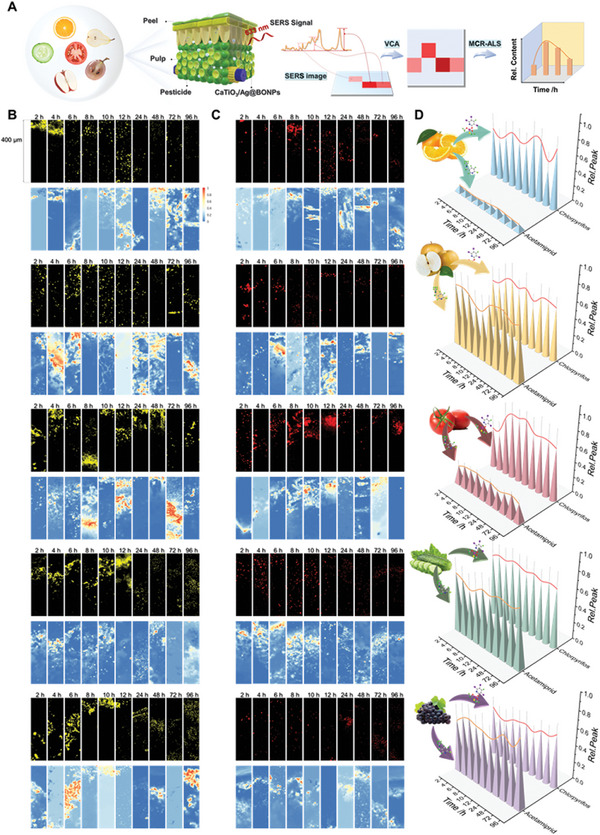
Pesticide penetration SERS imaging and related VCA and MCR‐ALS analyses results in various fruits and vegetables. A) Schematic representation of SERS imaging and data processing principles. SERS imaging (black background) detection and corresponding VCA analysis (blue background) of acetamiprid B) and chlorpyrifos C) at 2, 4, 6, 8, 10, 12, 24, 48, 72, and 96 h. D) Comparison of penetration content of non‐systemic pesticides and systemic pesticides in different fruits and vegetables (From top to bottom are oranges, pears, tomatoes, cucumbers, and grapes). Color lines represent mean changes obtained from a B‐spline curve fit.

In theory, the cuticle of fruits will show different resistance to different pesticide structures. Different kinds of fruits and vegetables were selected to explore application ability of this strategy in pesticide penetration, such as oranges with rough surfaces that need to be peeled, pears with smooth skin that can be eaten directly, grapes with black skin, and tomatoes with thin skin, and cucumbers with bumpy skin. The peels of these fruits and vegetables had different characteristics, and their flesh also had different degrees of fluorescence, which might affect the penetration of pesticides. Non‐systemic pesticide chlorpyrifos and systemic pesticide acetamiprid were sprayed on common fruits and vegetable surfaces for long‐term penetration monitoring within 400 µm (**Figure** **5**A). Representative SERS spectra of pesticides in different fruits and vegetables are shown in Figure S14 (Supporting Information). The SERS imaging results (Figure 5B,C) showed that pesticide penetration was deep in pears, tomatoes, and grapes, while shallow in cucumber. Differential penetration could be attributed to the fact that most pesticides were adsorbed on the epidermis of cucumber with less penetration, which might be related to the epidermal characteristics of cucumber. Acetamiprid was commonly permeated in various fruits and vegetables, especially tomatoes, cucumbers, and grapes, while chlorpyrifos was most permeable in tomatoes. The interferences, such as background fluorescence, were further removed with VCA. According to the results of the VCA analysis, the penetration ability of chlorpyrifos was weak, and the penetration depth was shallow. On the contrary, acetamiprid penetration was strong, especially in pears, tomatoes, and grapes, while the permeability in cucumbers was poor, which was consistent with the above study results.^[^ ^40^, ^41^, ^42^ ^]^ The differences between the VCA analysis results and SERS imaging results might be attributed to the fact that the flesh of fruits and vegetables selected in the experiment had high fluorescence and complex components, affecting the collection of SERS spectra to some extent. VCA analysis could extract various components from the pulp separately, identify the most similar spectrum to the pesticide according to the characteristic peaks, and convert the spectrum into a distribution heat map, which could effectively avoid the interference of other components and obtain a more accurate distribution of pesticides in the pulp. The SERS imaging and VCA analysis results of non‐systemic and systemic pesticides showed that the permeability of pesticides in the same fruit and vegetable was different, which might be related to the structure and action form of pesticides. Systemic pesticides can be absorbed by crops and penetrate deeper and long‐lasting when absorbed by crops. While non‐systemic pesticides are tactile pesticides, which mainly stay on the surface of crops and decrease with time. Moreover, the cuticle on the epidermis of fruits and vegetables is a natural barrier against fungi and pesticides. There are significant differences in the penetration of pesticides into different fruits and vegetables, which might be related to the different epidermal characteristics of fruits and vegetables and the ability of cuticles to resist pesticides. When the similarity is greater than 0.8 in VCA analysis, it is regarded as a pesticide. Then the permeability of acetamiprid was ≈200 µm, and some even exceeded 300 and close to 400 µm. However, the penetration depth of chlorpyrifos was ≈200 µm except in pears. While the position distribution of signals with similarity between 0.5 and 0.8 was closer to SERS imaging map and tended to be deeper, which was speculated to be a part of the metabolized and decomposed pesticide, however, specific evidence is needed for further verification. Overall, these results provided favorable evidence for the study of pesticide residues in fruits and vegetables. The penetration SERS imaging of pesticides on different crop profiles was quantified using MCR‐ALS, and the results are depicted in Figure 5D and Figure S15 (Supporting Information). The change of penetration contents of acetamiprid and chlorpyrifos was analyzed at 2, 4, 6, 8, 10, 12, 24, 48, 72, and 96 h. The quantization curve showed no significant difference in the permeability of chlorpyrifos between different fruits and vegetables, while acetamiprid had less penetration in oranges and tomatoes. The change curve of penetration contents indicated that the permeation content of pesticide may not be directly related to the action form of pesticide, and non‐systemic pesticides with high permeability may also penetrate into the pulp in quantities.^[^ ^43^, ^44^ ^]^ Therefore, fruits and vegetables using this type of non‐systemic pesticide are recommended to be eaten after peeling. The above results realized the relative quantification of pesticides permeated in various high‐fluorescence and high‐interference fruits and vegetables. Therefore, this method can also be extended and applied to 3D SERS imaging, which is expected to achieve quantitative analysis of the spatial range of pesticide penetration.

### 2.4 Establishment and Application of SML‐Based Pesticide Residue Identification System

**Figure 6:**
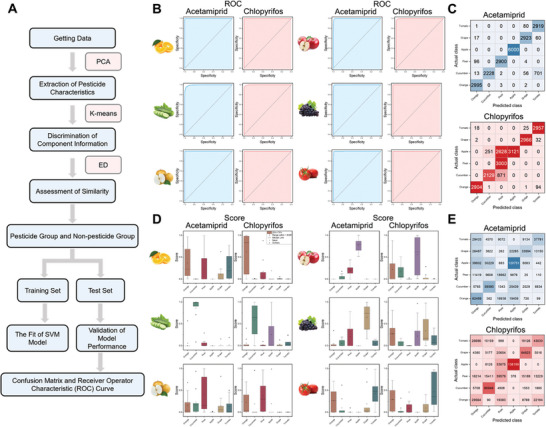
Spatial machine learning for SERS imaging of pesticide penetration. A) The computational flow of machine learning. B) ROC curves of the training set of acetamiprid and chlorpyrifos. C) Confusion matrix for acetamiprid and chlorpyrifos test samples. Application of the established SML method. D) Trends in scores of different fruits and vegetables. E) Confusion matrix for acetamiprid and chlorpyrifos.

Using K‐means cluster analysis, 140 000 spectra extracted from permeated SERS images of different fruits and vegetables were trained and analyzed to identify the pesticide spectra of acetamiprid and chlorpyrifos in different fruits and vegetables, and similarity was calculated with the spectra extracted by VCA to determine the data set for training (**Figure** **6**A), and the detailed procedure was described in Text S4 (Supporting Information). Multiple classifier models were trained with 70% spectral quantity, and the results are shown in Figure 6B and Figure S16 (Supporting Information). The area under the curve (AUC) of receiver operating characteristic (ROC) was >0.92 (Tables S2 and S3, Supporting Information), and a classifier with good discriminatory performance was obtained. Using the remaining 30% of the untrained spectra for prediction, and the confusion matrix in Figure 6C shows good prediction results. The established method is then applied to a total of 1.3 million spectra extracted from permeated SERS imaging samples, which includes six kinds of fruit and vegetable types. The pesticide proportional score graph obtained by counting the spectral proportional scores of the various fruits and vegetables identified showed that for the samples tested, most of the fruit and vegetable samples scored significantly higher in the corresponding fruit and vegetable categories (Figure 6D). The results of the classification of acetamiprid and chlorpyrifos for all tested samples according to this decision rule were summarized in a confusion matrix (Figure 6E), which shows that this method will be able to identify pesticide residues in different categories of fruits and vegetables, further indicating that it can be used as a tool for pesticide residue analysis.

Combining space machine learning with artificial intelligence, the model established by this method has a good ability to identify pesticides in different fruits and vegetables with an area under the ROC line as high as 0.92. From the score trends and confusion matrix plots, it can be seen that SML method can correctly identify pesticides from a large amount of SERS imaging information and discriminate them with VCA results. This method combines unsupervised machine learning and supervised machine learning with the VCA results to correctly predict pesticides in fruits and vegetables, improve the prediction accuracy, and provide a more scientific and accurate method for pesticide residue detection.

### 2.5 Expansion of Penetration Detection for Mixed Pesticides

**Figure 7:**
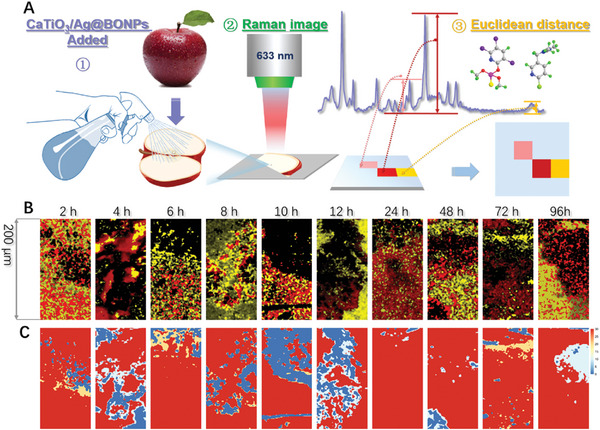
The penetration behavior of mixed pesticides in apples. A) Illustrates the visualization process of mixed pesticide infiltration. B) Displays pesticide penetration SERS imaging of chlorpyrifos (highlighted in red) and acetamiprid (highlighted in yellow) within 200 µm at 2, 4, 6, 8, 10, 12, 24, 48, 72, and 96 h. C) Shows the results of Euclidean distance (ED) analysis for the corresponding pesticides.

Exploring the mixed penetration will have guiding significance for the combined use of pesticides in actual production. The preliminary exploration of mixed pesticide penetration was carried out by spraying acetamiprid and chlorpyrifos mixture on apples and compared with that of the single pesticide application (Figure 4B,E). The specific process is shown in **Figure** **7**A. The characteristic peak of a single pesticide was found in the mixed spectrum (Figure S17, Supporting Information). Further, SERS imaging was used to distinguish the distribution positions of different pesticides in the mixture within 200 µm at 2, 4, 6, 8, 10, 12, 24, 48, 72, and 96 h. The similarity was calculated by ED and compared with SERS imaging to obtain the penetration distribution of each pesticide and overlapping position distribution of pesticide mixture. The color value of the position without pesticides was set to 0, the color value of chlorpyrifos on the heat map was set to 10, and the color value of acetamiprid was set to 20. The color value of the overlapping part was the addition of corresponding color value. As depicted in Figure 7B,C, the synergic penetration of chlorpyrifos and acetamiprid within 200 µm showed that the overall penetration degree of the mixed pesticide is more distributed and deeper than that of the individual. Therefore, in order to ensure the effect of pesticides and reduce toxicity, interval application can be considered in the combined application of pesticides. The specific principle of the interaction of mixed pesticides in the permeation process needs to be further studied, and our future research will continue to focus on this aspect.

## 3 Conclusion

In conclusion, this study introduces an innovative visual monitoring strategy, CaTiO_3_/Ag@BONPs‐SERS‐AI, for pesticide molecular analysis within plants. The integration of machine learning methodologies with 3D and dynamic SERS imaging technology has led to a breakthrough in the visualization of pesticide penetration behavior and the removal of penetrated pesticide molecules within crops. By employing multivariate data processing and analysis, we were able to extract highly detailed information from complex Raman spectra, facilitating the visualization and quantification of pesticide molecule permeation across plant compartments. Through meticulous analysis utilizing the Diversity Data Module, we unveil a previously undisclosed weakening process during pesticide penetration and the remarkably high permeability observed in non‐systemic pesticides, challenging conventional theories. Furthermore, our findings raise concerns regarding potential interactions resulting from the combined application of multiple pesticides. Collectively, not only does this work offer a comprehensive framework for assessing the behavior of pesticide molecules in plant systems, but it also paves the way for optimizing pesticide application strategies and controlling crop growth safety undertaking. Furthermore, the novel application of machine learning to Raman imaging holds significant promise for enhancing the resolution and interpretability of complex spectral data, with potential applications in various fields beyond plant biology.

## 4 Experimental Section

### Chemicals and Reagents

Silver nitrate (AgNO_3_, 99.9%) was supplied by Alfa Aesar (China). Sodium borohydride (NaBH_4_, 99.99%) was procured from Sigma‐Aldrich© (St. Louis, USA). Perovskite (CaTiO_3_, 99.5%) was obtained from Shanghai Macklin Biochemical Co., LTD. Methanol (analytical reagent), thiabendazole, thiram, acetamiprid, and chlorpyrifos were sourced from Aladdin Chemical Co., Ltd (Shanghai, China). Apples, grapes, pears, oranges, cucumbers, and tomatoes were purchased from local supermarkets.

### CaTiO_3_/Ag@BONPs Preparation

CaTiO_3_/Ag@BONPs were prepared by the reduction of silver nitrate with sodium borohydride and the subsequent addition of CaTiO_3_ at room temperature. In a 3‐necked flask, 10 mL of NaBH_4_ (0.0175 m) was mixed with 480 mL of water and stirred at a rate of 1500 r min^−1^. After 10 min, 10 mL of AgNO_3_ (0.0195 m) was added, causing the solution to change to a yellowish‐green color. The stirring rate was increased to 2200 r min^−1^, and the solution was stirred for an additional 15 min. Subsequently, the resulting solution containing silver nanoparticles (Ag@BONPs) was centrifuged at 5200 r min^−1^ for 20 min. The supernatant was discarded, and CaTiO_3_ solid was incubated with Ag@BONPs. This led to the formation of the novel multifunctional SERS substrate, CaTiO_3_/Ag@BONPs (refer to Figure 2A).

### Solution Preparation

Pesticide standards (chlorpyrifos, thiabendazole, thiram, and acetamiprid) were diluted with methanol to make a pesticide storage solution of 1000 mg L^−1^. These prepared standard solutions were stored at 4 °C in the dark and diluted to low concentrations with purified water for application.

### Sample Preparation

Fresh samples underwent a thorough rinsing process with tap water and methanol, followed by washing with Wahaha water, and were subsequently left to air dry naturally. Subsequently, pesticides were applied to the surfaces of blank apples, grapes, pears, oranges, cucumbers, and tomatoes to replicate real‐world conditions. These treated samples were then allowed to air dry naturally for future use.

### SERS Measurement

SERS analysis was conducted using a Raman spectrometer manufactured by WITec in Germany. Pesticide detection was carried out on longitudinally sectioned fruits and vegetables, following the direction of the peel. The accumulation time for SERS spectrum acquisition was set at 3 s, while imaging was performed with a 0.1‐s exposure. Correspondingly, the power for SERS detection was configured at 3 mW, and for imaging, it was set at 0.5 mW. The dimensions of the 3D SERS imaging were maintained at 50 µm for length, width, and depth, while the SERS dynamic imaging featured a length of 100 µm and a width of 50 µm. The laser power was maintained at 3 mW, and the scanning time was 0.05 s. The laser operated at a wavelength of 633 nm, and a single scanning pass was executed. The temperature was held constant at 25 °C. Further details of the operational procedure can be found in Movie S3 (Supporting Information).

### Data Analysis

The raw data was processed with baseline correction using the software LabSpec (Figure S18, Supporting Information). Data analysis was conducted utilizing R language and Origin 2021. SERS imaging data underwent analysis employing vertex component analysis, multivariate curve resolution alternate least squares, K‐means cluster analysis, and Euclidean distance calculations. The color speckle images were imported into ImageJ, an open‐source image processing software, to generate 3D images and dynamic SERS imaging movies.

## Conflict of Interest

The authors declare no conflict of interest.

## Author Contributions

X.W. and X.S. contributed equally to this work. X.W. performed conceptualization, methodology, and data curation, and wrote the original draft; X.S. performed methodology, visualization, investigation, and writing original draft; Z.L. performed methodology and data curation; Y.Z. performed data curation; G.W. performed data curation; Y.W. performed methodology and supervision; Q.L. performed methodology and supervision; C.Y. performed methodology and supervision; T.B. performed methodology and supervision; Y.L. performed methodology and supervision; J.H. performed methodology and supervision; Y.L. wrote and reviewed and edited the final manuscript.

## Supporting information

Supporting Information

Supplemental Movie 1

Supplemental Movie 2

Supplemental Movie 3

## Data Availability

The data that support the findings of this study are available in the supplementary material of this article.
