# Supplementary material for: Surface‐Enhanced Raman Scattering Imaging Assisted by Machine Learning Analysis: Unveiling Pesticide Molecule Permeation in Crop Tissues
Source: Adv Sci (Weinh). 2024 Jun 24;11(32):2405416. doi: 10.1002/advs.202405416 (PMC11347994; doi:10.1002/advs.202405416)
Supplement: Supplementary file 1 — Supporting Information [file ADVS-11-2405416-s003.pdf]

ADVANCED SCIENCE
Open Access

Supporting Information

for *Adv. Sci.*, DOI 10.1002/advs.202405416

Surface-Enhanced Raman Scattering Imaging Assisted by Machine Learning Analysis: Unveiling Pesticide Molecule Permeation in Crop Tissues

*Xiaotong Wang, Xiaomeng Sun, Zhehan Liu, Yue Zhao, Guangrun Wu, Yunpeng Wang, Qian Li, Chunjuan Yang, Tao Ban, Yu Liu, Jian-an Huang and Yang Li**

Supplementary Materials

Revolutionizing Raman Imaging: Unveiling Drug Molecule Permeation in Plant Tissues through Deep Learning Analysis

*Xiaotong Wang [#], Xiaomeng Sun[#], Zhehan Liu, Yue Zhao, Guangrun Wu, Yunpeng Wang, Qian Li, Chunjuan Yang, Tao Ban, Yu Liu, Jian-an Huang, Yang Li**

Xiaotong Wang, Xiaomeng Sun, Yue Zhao, Guangrun Wu, Yunpeng Wang, Qian Li, Chunjuan Yang, Yang Li
Research Center for Innovative Technology of Pharmaceutical Analysis (State Key Laboratory of Frigid Zone Cardiovascular Diseases), College of Pharmacy, Harbin Medical University, Heilongjiang 150081, PR China.

Zhehan Liu
College of Bioinformatics Science and Technology, Harbin Medical University, Heilongjiang 150081, PR China.

Tao Ban
Department of General Surgery, The Fourth Affiliated Hospital of Harbin Medical University, and Department of Pharmacology (State Key Laboratory of Frigid Zone Cardiovascular Diseases, Ministry of Science and Technology; The Key Laboratory of Cardiovascular Research, Ministry of Education) at College of Pharmacy, Harbin Medical University, Baojian Road, Nangang District, Harbin 150081, PR China.

Yu Liu
Department of Clinical Laboratory Diagnosis, Fourth Affiliated Hospital of Harbin Medical University, Harbin, China

Yang Li, Jian-an Huang
Research Unit of Health Sciences and Technology (HST), Faculty of Medicine University of Oulu, Finland.
E-mail: liy@hrbmu.edu.cn, Yang.Li@oulu.fi (Yang Li)

S1. Substrate performance verification and characterization

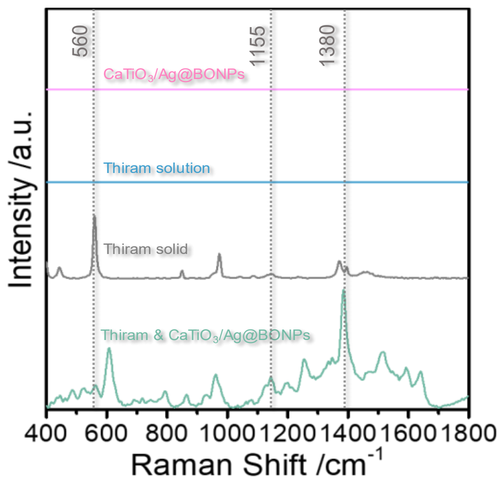

Figure S1. *Validation of CaTiO$_3$/Ag@BONPs for the detection of thiram.*

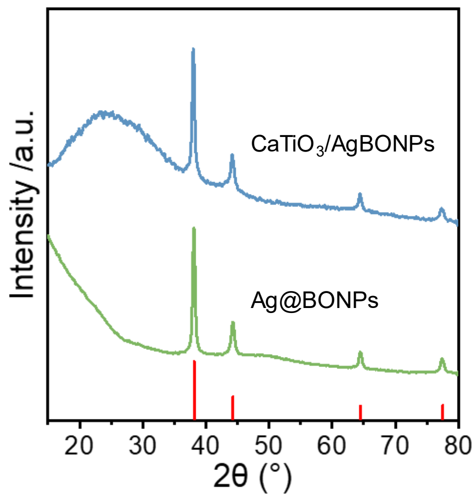

Figure S2*. X-ray diffraction (XRD) of the CaTiO$_3$/Ag@BONPs (blue line) and Ag@BONPs (green line).*

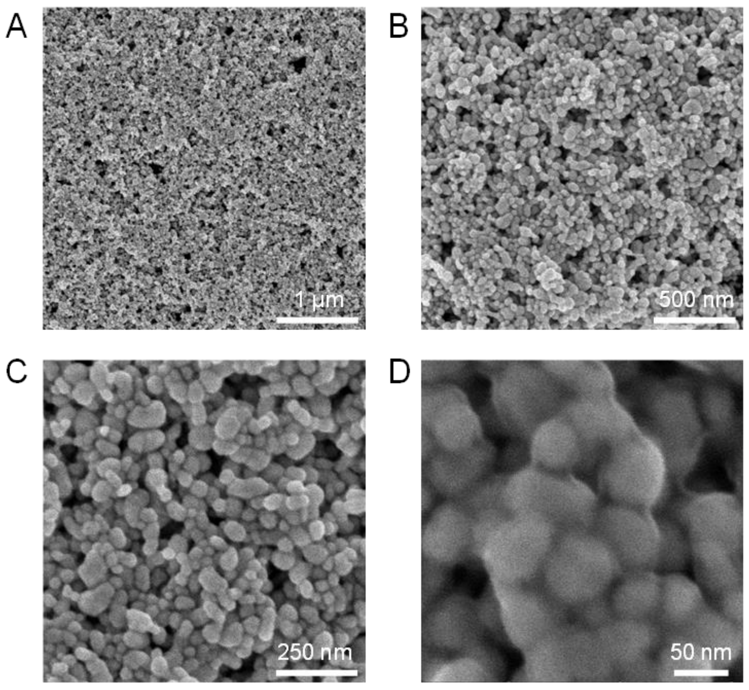

Figure S3*. Scanning electron microscope (SEM) of the CaTiO$_3$/Ag@BONPs at 1 μm (A), 500 nm (B), 250 nm (C) and 50 nm (D) in the sprayed state.*

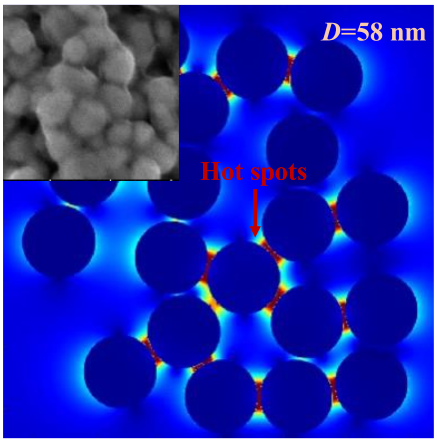

***Figure S4**. The corresponding FDTD simulation according to the substrate distribution in SEM.*

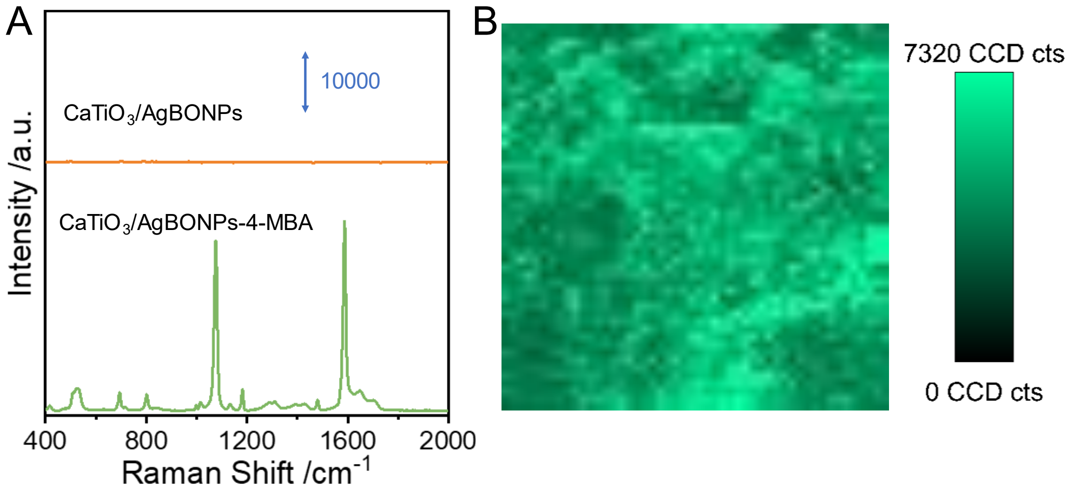

***Figure S5**. A. SERS spectra of $CaTiO_3$/Ag@BONPs (yellow line) and $CaTiO_3$/Ag@BONPs-4-MBA (green line). B. SERS imaging of $CaTiO_3$/Ag@BONPs-4-MBA distribution on pulp.*

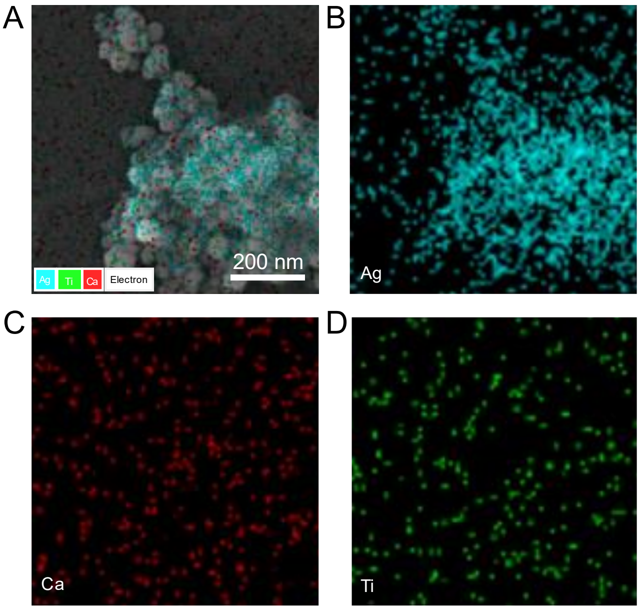

Figure S6. *A. Scanning electron microscope (SEM) and Energy Dispersive Spectrometer (EDS) mapping of the $CaTiO_3$/Ag@BONPs in the sprayed state. The respective distributions of silver (B), calcium (C), and titanium (D) elements.*

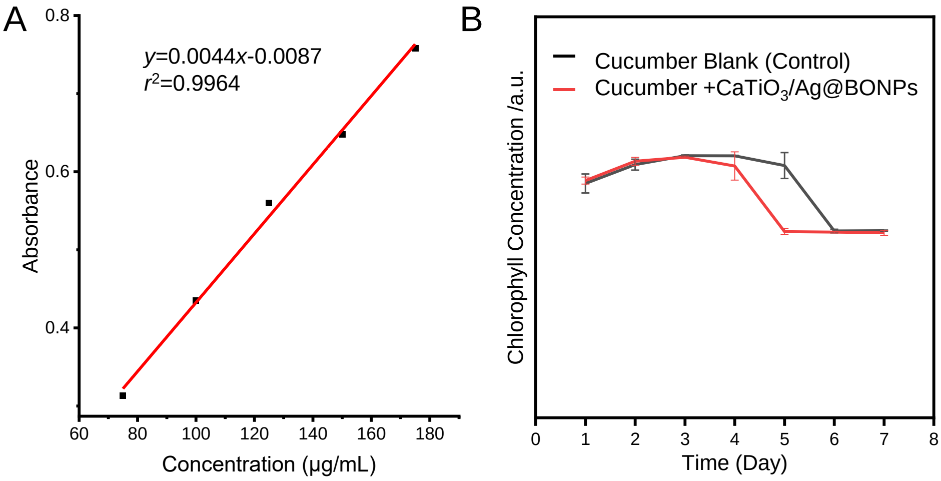

Figure S7. *The chlorophyll content in cucumber after spraying with $CaTiO_3$/Ag@BONPs. A. Standard curves for UV absorption of chlorophyll. B. Comparison of chlorophyll concentrations. The data are mean ± SD from three individual experiments.*

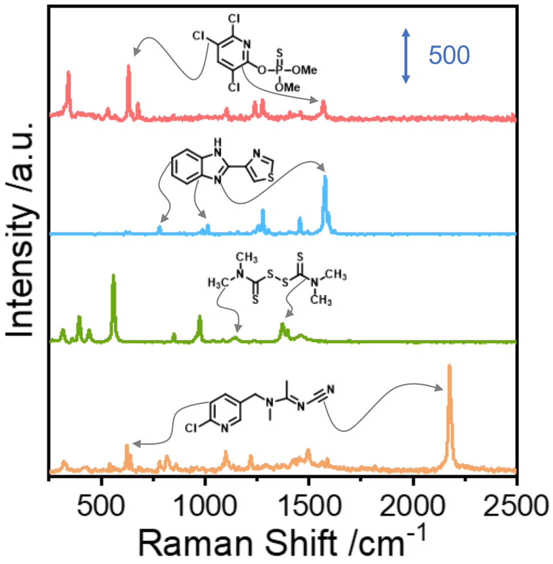

Figure S8. *Raman signals of chlorpyrifos (red line), thiabendazole (blue line), thiram (green line), and acetamiprid (yellow line) standard solids.*

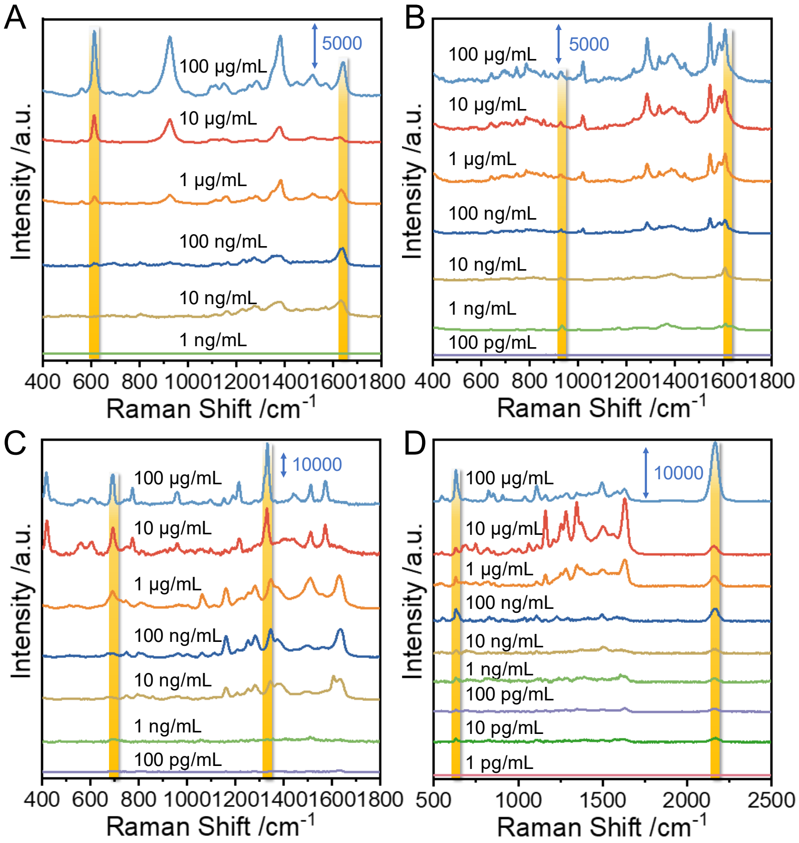

Figure S9. *Lower limits of detection of different pesticides. SERS spectra of chlorpyrifos (A), thiabendazole (B), thiram (C) and acetamiprid (D) at different concentrations.*

Text S1: Calculation of the Enhancement Factor (*EF*) for thiram

The enhancement factor (*EF*) of thiram can be calculated as[1]:

$$EF = (I_{SERS} / I_{Raman}) \times (N_{Raman} / N_{SERS})$$

I_{SERS} was the peak intensity of the 1380 cm^{-1} peak in the SERS spectrum and I_{Raman} was the peak intensity of the same vibrational mode.

Thiram with a mass of 0.0005 g were taken for Raman test. 2.5 μL pesticide were added to the 5 μL Ag@BONPs, and were absorbed by a capillary with an inner diameter of 0.5 mm. SERS detection was performed under a 10-fold mirror, and the laser diameter was 1545 nm.

Both of these spectra were recorded using a 633 nm laser under the same conditions and the exposure time was set to 3 s with a one-time accumulation.

C_{SERS}=0.01×2.5/ (2.5+5) =3×10^{-3} mg/mL =3×10^{-6} g/mL

$V_{SERS} = (0.025)^2\times\pi\times1.545\times10^{-4} = 3\times10^{-7}$ cm^3

$$N_{SERS} = C_{SERS}V_{SERS} \times N_A = 5.5\times10^{11}$$

Then, the number of thiram molecules being illuminated in the normal Raman detection:

$$N_{Raman} = m_{Raman} \times N_A/M = 1.2\times10^{18}$$

From the above parameters,

$EF = (I_{SERS} \times N_{Raman}) / (I_{Raman} \times N_{SERS}) = (14071 \times 1.2\times10^{18}) / (272\times5.5\times10^{11}) = 1.1 \times 10^8$

Based on the same process, the calculations of the Ag@BONPs to thiabendazole, chlorpyrifos and acetamiprid are as follows:

thiabendazole: 2.1×10^7; chlorpyrifos: 3.5×10^8; acetamiprid: 1.0×10^8

The calculations of the $CaTiO_3$/Ag@BONPs to thiram, thiabendazole, chlorpyrifos and acetamiprid are as follows:

thiram: 2.8×10^8; thiabendazole: 4.2×10^7; chlorpyrifos: 1.2×10^9; acetamiprid: 3.7×10^8

Text S2: FDTD principle and simulation process

According to the previous DLS result, the diameter of Ag@BONPs was set to be 58 nm, the diameter of $CaTiO_3$/Ag@BONPs was set to be 110 nm and the nanogap between adjacent nanoparticles was set to be 2 nm. The surrounding medium was set to be 1.0 (n_{medium} = 1.0). Simulated result was shown in Figure 2B, it could be seen that there are hot spots between silver nanoparticles, which was shown with red arrow, and the maximum electric field intensity of the sample was about 36.7 V/m.

According to the EF_{EM} calculation formula,

$$EF = \frac{|E_{out}|^2 |E'_{out}|^2}{|E_0|^4}$$

where E_0 is the incident electric field intensity, E_0 = 1 V/m, and E_{out} is the electric *field intensity of the position of hot spots caused by the incident light, E'_{out} refers to the* field evaluated at the scattering frequency. The local electric field intensity of SERS ($|E_{out}|^2 \times |E'_{out}|^2$) is approximately equal to the surface localized electric field intensity of nanoparticles ($|E_{out}|^4$). Therefore, the above formula can be changed into $EF_{EM} = \frac{|E_{out}|^4}{|E_0|^4}$. For D_{Ag}= 58 nm, $EF_{EM} = 36.7^4 \approx 1.8 \times 10^6$.

Simulated result was shown in Figure 2B, it could be seen that there are hot spots, which were shown with red arrow. For D_{Ag}= 110 nm, $EF_{EM} = 127^4 \approx 2.6 \times 10^8$. From the calculated results of EF_{EM}, it could be found that the electromagnetic field enhancement effects of Ag@BONPs and $CaTiO_3$/Ag@BONPs are extremely obvious, which is consistent with the experimental trend.

S2. Comparison of detection methods

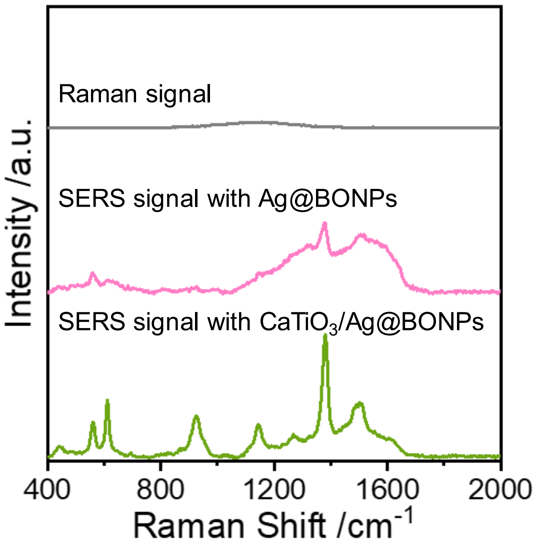

Figure S10. *Comparison of the current method with conventional methods in signal. Raman signal of thiram in pulp (gray line) and SERS signal using Ag@BONPs (pink line) and CaTiO$_3$/Ag@BONPs (green line) substrates.*

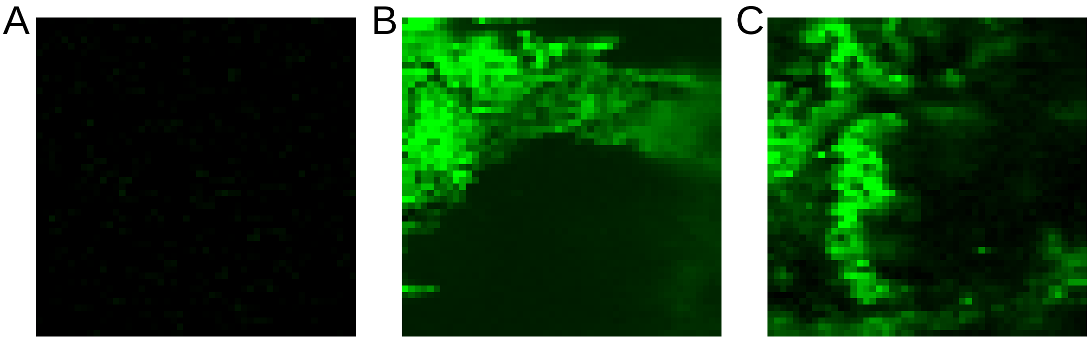

Figure S11. *Comparison of the current method with conventional methods in imaging. Raman imaging of thiram in pulp (A) and SERS imaging using Ag@BONPs (B) and CaTiO$_3$/Ag@BONPs (C) substrates.*

S3. Comparison of elution methods

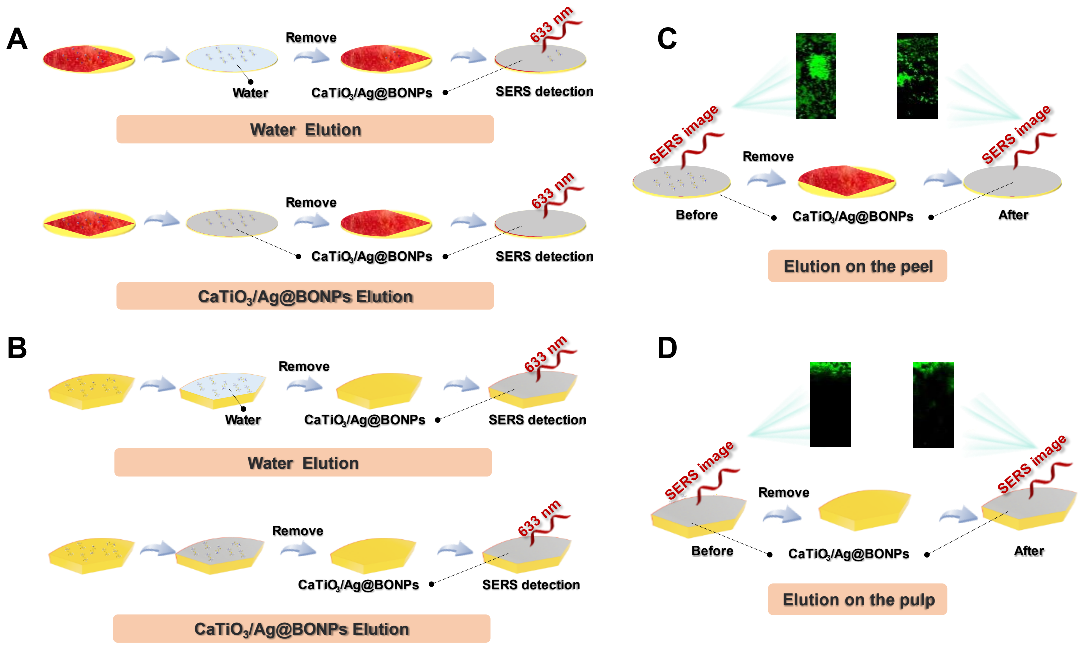

Figure S12. *Specific steps of elution. A. The process of water and $CaTiO_3$/Ag@BONPs elution from the pericarp. B. The process of water and $CaTiO_3$/Ag@BONPs elution from the pulp. C. Processes before and after $CaTiO_3$/Ag@BONPs elution from the pericarp. D. Processes before and after $CaTiO_3$/Ag@BONPs elution from the pulp.*

S4. The Raman peak assign to pesticides

Table S1: The bands and their assignment appeared in the SERS spectra of pesticides.

Pesticides	Raman shift (cm^{-1})	Band assignments	**Ref**
Thiabendazole	640	δC-C-C δS-C-N	[2-7]
	784	γC-H	[2-7]
	1019	βC-H	[2-7]
	1285	νR	[2-7]
	1544	νC=N	[2-7]
	1577	νC=N	[2-7]
Thiram	560	νS-S	[2, 4, 6, 8]
	925	νCH_3-N νC=S	[2, 4, 6, 8]
	1155	ρCH_3 νC-N	[2, 4, 8]
	1380	δCH_3 νC-N	[2, 4, 8]
	1514	ρCH_3 νC-N	[2, 4, 8]
Chlorpyrifos	416	νP-O-P	[4, 6, 9-11]
	602	δC=O	[4, 6, 9-11]
	668	δP=S	[4, 6, 9-11]
	687	νC-Cl	[4, 6, 9-11]
	772	νP-S δCH_2	[6, 9-11]
	1023	νP-O-C	[4, 6, 9-11]
	1184	δC-N	[4, 6, 9-11]
	1208	Cl-R, δ(C–H)	[4, 6, 9-11]
	1331	Cl-R, δ(C–H), ν(C=C)	[4, 6, 9-11]
	1568	νR	[4, 6, 9-11]
Acetamiprid	442	ν(C=C)	[6, 11-15]
	633	ρR	[6, 11-15]
	826	ρR	[6, 11-15]

	1033	brR	[6, 11-15]
	1107	brR	[6, 11-15]
	1496	νR	[6, 11-15]
	1566	ν(C=C)	[6, 11-15]
	2160	δC-N	[6, 11-15]

Note: br, breathing; R, ring; β, in-plane bending; γ, out-of-plane bending; δ, deformation; ν, stretching; ρ, rocking.

S5. Quantification of pesticide permeation

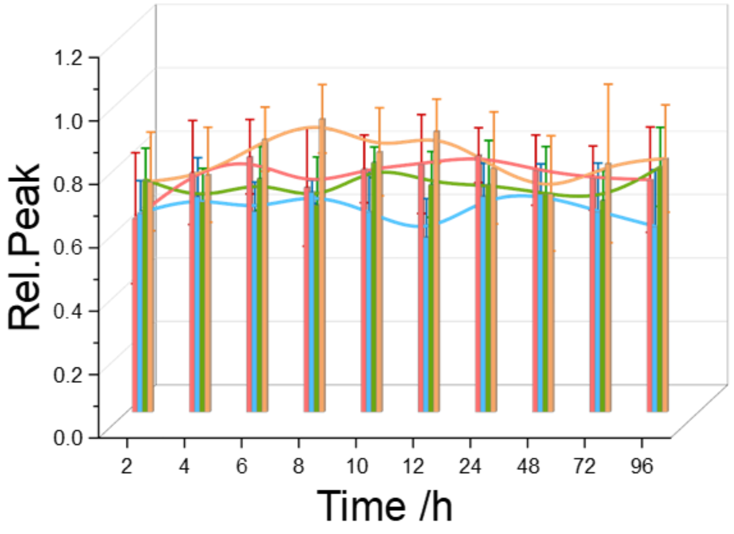

Figure S13. *The change of penetration content of chlorpyrifos (red), thiabendazole (blue), thiram (green), and acetamiprid (yellow) was obtained at 2, 4, 6, 8, 10, 12, 24, 48, 72, and 96 h. Colored lines represent mean changes obtained from a B-spline curve fit.*

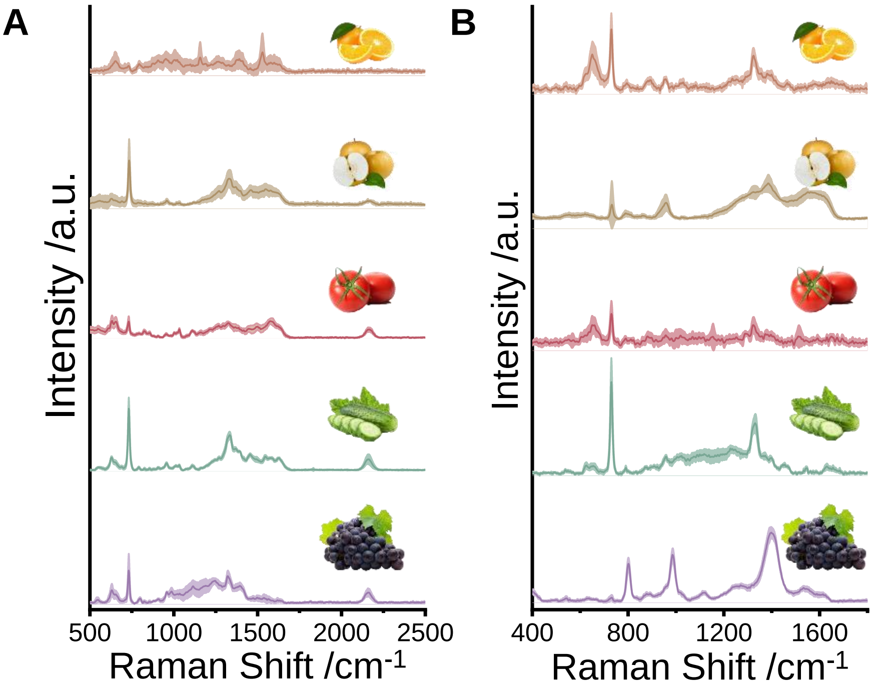

Figure S14. *Representative SERS spectra of acetamiprid and chlorpyrifos in fruits and vegetables.*

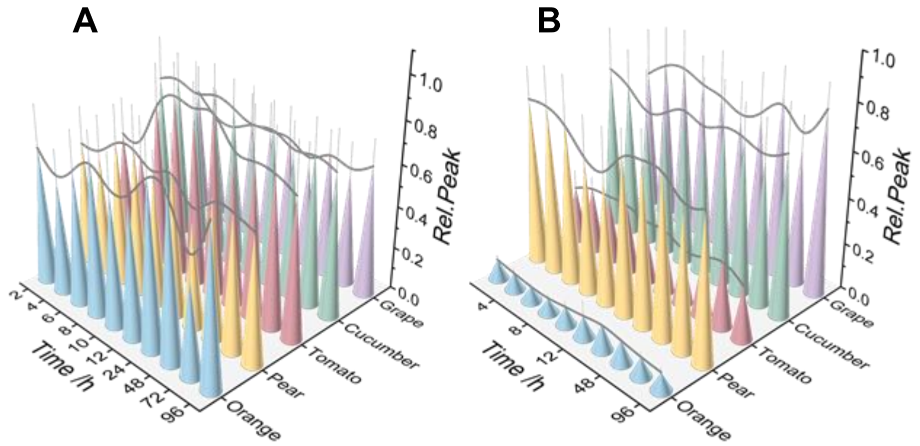

Figure S15. *The change of penetration content of chlorpyrifos (A) and acetamiprid (B) was obtained at 2, 4, 6, 8, 10, 12, 24, 48, 72, and 96 h.*

S6. Machine learning

Text S3: Detailed process of machine learning

Firstly, the Raman spectrum data of pesticides were read, and the dimensionality of the data was reduced according to the distance of 10 sites before and after the characteristic peak interval of the pesticide. In addition, in order to extract the features of pesticides more deeply, the "procomp" function in R language was used for principal component analysis (PCA), and the new features were combined to describe the changes of feature peak position and peak intensity. The two principal components with the largest cumulative variance contribution rate were PC1 and PC2. The R language "ggplot" function was used to visualize and compare the Raman spectral data of multiple groups of samples using the eigenvector of the covariance matrix. The Raman spectra are projected to a fraction plot in proportion to the load, the PCA 2D fraction plot, juxtaposed with a 95% confidence ellipse. In order to effectively distinguish the component information in the spectral image, the "fviz_nbclust" function of R language was used to view the best k value of the best K-means clustering, and then the "kmeans" function was called to complete the clustering. From the degree of boundary between pesticides in the visualization results, the clustering module had good intra-group similarity and inter-group difference. In order to measure which module is most likely to be the pesticide part, the "pure pesticide" data is used to evaluate the similarity of all channel results in each K-means clustering module in the image. The evaluation method is to call the similarity measure based on Euclidean distance of the R language "matchFactor" function. Thus, the data as pesticide group and non-pesticide control group were selected. The obtained data were divided into training set and test set, and then the R language "svm" function was used to fit the SVM model based on the training data set, and the performance of the model was verified on the test data set, that is, the confusion matrix and ROC curve were output.

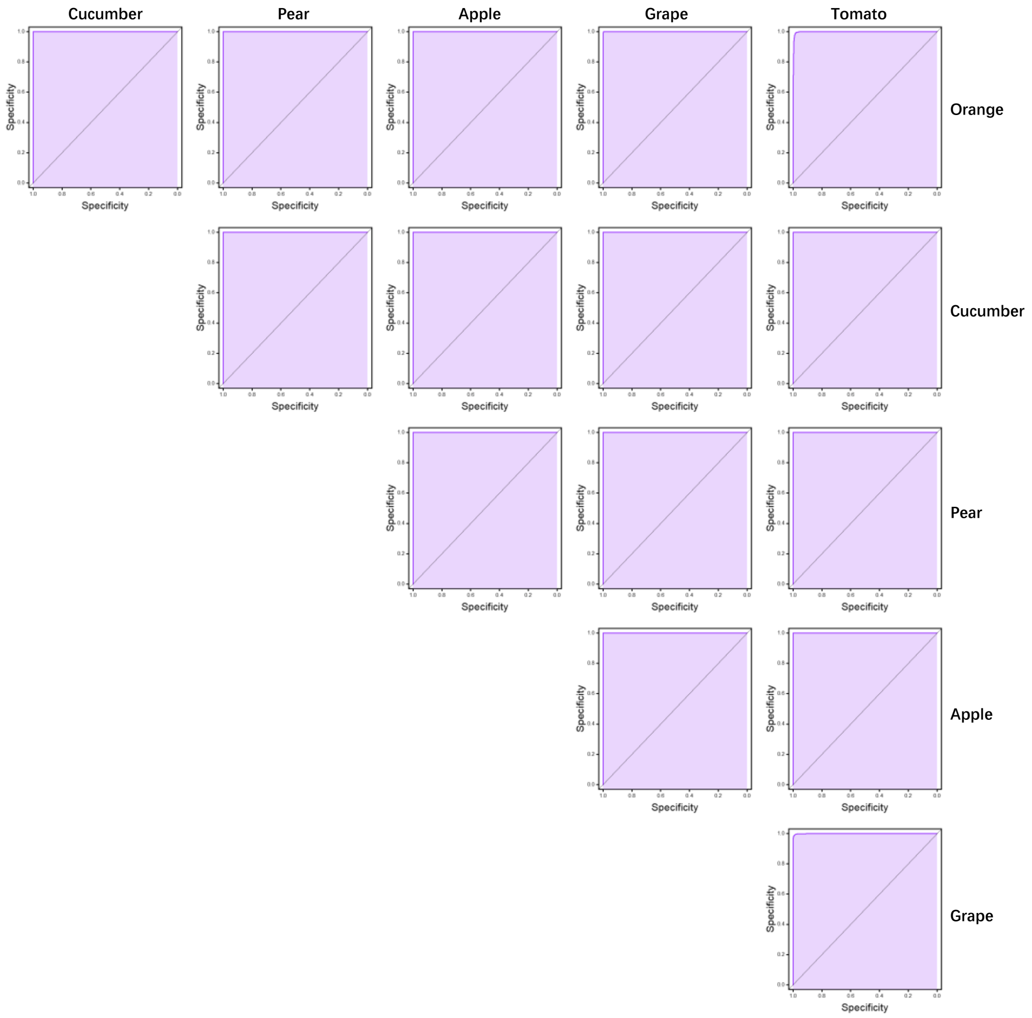

Figure S16. *ROC curves between different fruits and vegetables.*

Table S2: The AUC values of acetamiprid ROC curves.

	Orange	Cucumber	Pear	Apple	Grape	Tomato	Acetamiprid
Orange	-	0.999	0.980	1.000	0.995	1.000	0.998
Cucumber	-	-	0.959	0.991	0.985	0.990	0.937
Pear	-	-	-	0.992	0.987	0.991	0.927
Apple	-	-	-	-	0.988	0.991	0.970
Grape	-	-	-	-	-	0.969	0.980
Tomato	-	-	-	-		-	0.994
Acetamiprid	-	-	-	-	-	-	-

Table S3: The AUC values of chlorpyrifos ROC curves.

	Orange	Cucumber	Pear	Apple	Grape	Tomato	Chlorpyrifos
Orange	-	0.960	0.960	0.960	0.960	0.977	0.959
Cucumber	-	-	0.999	0.977	0.999	0.995	0.965
Pear	-	-	-	0.977	0.999	0.995	0.984
Apple	-	-	-	-	0.999	0.995	0.980
Grape	-	-	-	-	-	0.985	0.996
Tomato	-	-	-	-		-	0.993
Chlorpyrifos	-	-	-	-	-	-	-

S7. Detection of penetration of mixed pesticides

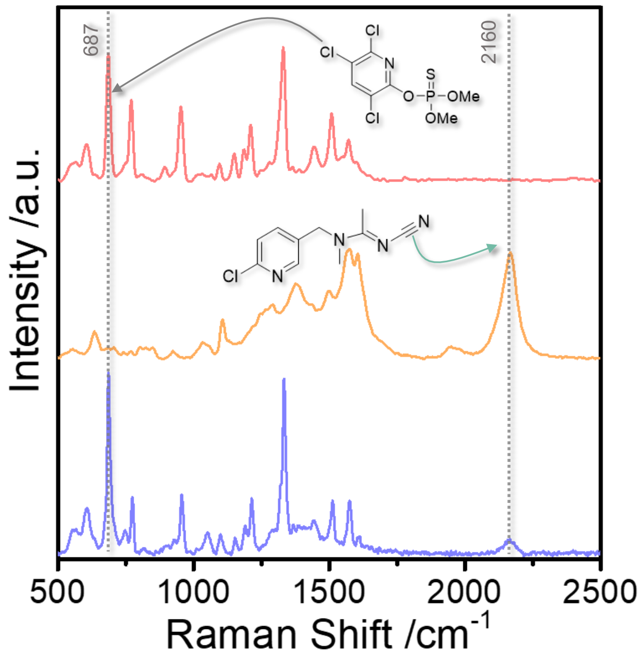

Figure S17. *Mixed SERS spectra (yellow line) of chlorpyrifos (grey line) and acetamiprid (green line) in pulp.*

S8. Correction of base

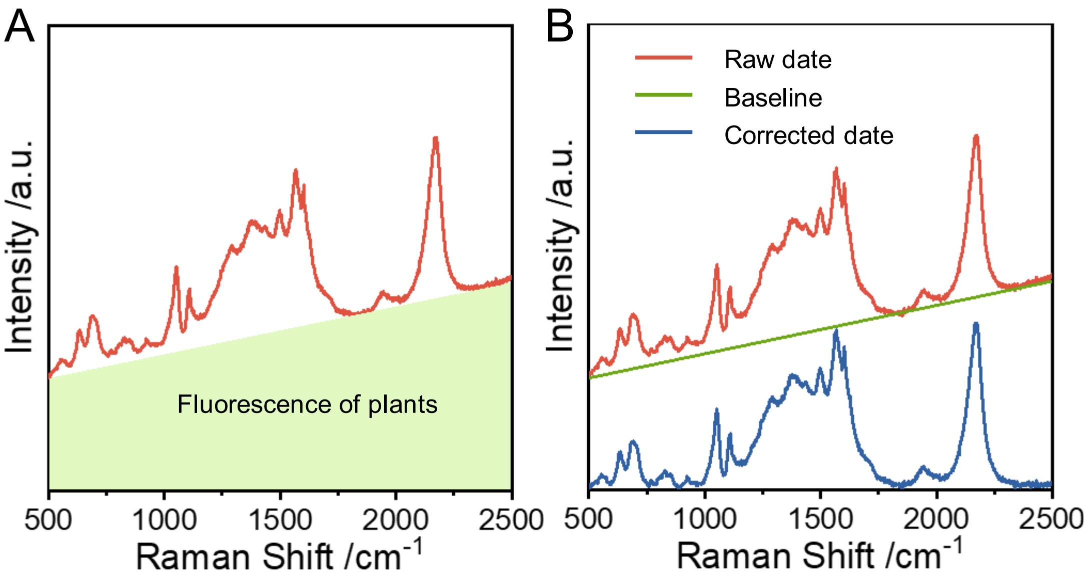

Figure S18. *Baseline correction for SERS spectra in plants. A. SERS spectra with plant fluorescence. B. Comparison of SERS spectra before and after correction.*

S9. References

[1] T. Xie, Z. Cao, Y. Li, Z. Li, F.-L. Zhang, Y. Gu, C. Han, G. Yang, L. Qu, *Food Chem.* **2022**, *381*, 132208.

[2] W. A. Tegegne, W.-N. Su, M.-C. Tsai, A. B. Beyene, B.-J. Hwang, *Applied Materials Today* **2020**, *21*, 100871.

[3] F. K. Alsammarraie, M. Lin, A. Mustapha, H. Lin, X. Chen, Y. Chen, H. Wang, M. Huang, *Food Chem.* **2018**, *259*, 219.

[4] S.-Y. Wang, X.-C. Shi, G.-Y. Zhu, Y.-J. Zhang, D.-Y. Jin, Y.-D. Zhou, F.-Q. Liu, P. Laborda, *Trends Food Sci. Technol.* **2021**, *116*, 583

[5] Z. Chen, Y. Sun, J. Shi, W. Zhang, X. Zhang, X. Huang, X. Zou, Z. Li, R. Wei, *Food Chem.* **2022**, *370*, 131276.

[6] T. Wang, S. Wang, Z. Cheng, J. Wei, L. Yang, Z. Zhong, H. Hu, Y. Wang, B. Zhou, P. Li, *Chem. Eng. J.* **2021**, *424*, 130323.

[7] P. Liou, F. X. Nayigiziki, F. Kong, A. Mustapha, M. Lin, *Carbohydr. Polym.* **2017**, *157*, 643.

[8] J. Zhu, S. Zhang, G.-j. Weng, J.-j. Li, J.-w. Zhao, *Spectrochimica Acta Part A: Molecular and Biomolecular Spectroscopy* **2021**, *262*, 120108.

[9] P. Ma, L. Wang, L. Xu, J. Li, X. Zhang, H. Chen, *Eur. Food Res. Technol.* **2020**, *246* (1), 239.

[10] X. Du, P. Wang, L. Fu, H. Liu, Z. Zhang, C. Yao, *Anal. Lett.* **2020**, *53* (6), 821.

[11] C. Zhai, Y. Peng, Y. Li, K. Chao, *J. Raman Spectrosc.* **2017**, *48* (3), 494.

[12] Y. Kang, T. Wu, X. Han, H. Gu, X. Zhang, *Microchimica Acta* **2018**, *185* (11), 504.

[13] Z. F. Gao, Y. X. Li, L. M. Dong, L. L. Zheng, J. Z. Li, Y. Shen, F. Xia, *Sensors Actuators B: Chem.* **2021**, *348*, 130728.

[14] C.-P. Yang, W.-Y. Kao, S.-H. Yu, F.-D. Mai, H.-Y. Tsai, J.-W. Chen, Y.-C. Liu, *Sensors Actuators B: Chem.* **2023**, *374*, 132798.

[15] M. M. Hassan, M. Zareef, T. Jiao, S. Liu, Y. Xu, A. Viswadevarayalu, H. Li, Q. Chen, *Food Chem.* **2021**, *338*, 127796.
